# Supplementary material for: Prevalence of high screen time and associated factors among students: a cross-sectional study in Zhejiang, China
Source: BMJ Open. 2018 Jun 19;8(6):e021493. doi: 10.1136/bmjopen-2018-021493 (PMC6009552; doi:10.1136/bmjopen-2018-021493)
Supplement: Author's manuscript [file bmjopen-2018-021493.draft_revisions.pdf]

BMJ Open is committed to open peer review. As part of this commitment we make the peer review history of every article we publish publicly available.

When an article is published we post the peer reviewers' comments and the authors' responses online. We also post the versions of the paper that were used during peer review. These are the versions that the peer review comments apply to.

The versions of the paper that follow are the versions that were submitted during the peer review process. They are not the versions of record or the final published versions. They should not be cited or distributed as the published version of this manuscript.

BMJ Open is an open access journal and the full, final, typeset and author-corrected version of record of the manuscript is available on our site with no access controls, subscription charges or pay-per-view fees (http://bmjopen.bmj.com).

If you have any questions on BMJ Open's open peer review process please email info.bmjopen@bmj.com

BMJ Open

Prevalence of high screen time and associated factors among Chinese adolescents

Journal:	*BMJ Open*
Manuscript ID	bmjopen-2018-021493
Article Type:	Research
Date Submitted by the Author:	03-Jan-2018

Complete List of Authors:	Wang, Hao; Zhejiang Provincial Center for Diseases Control and Prevention, Department of NCDs Control and Prevention<br>Zhong, Jie-Ming; Zhejiang Provincial Center for Diseases Control and Prevention, Department of NCDs Control and Prevention<br>Hu, Ru-Ying; Zhejiang Provincial Center for Diseases Control and Prevention, Department of NCDs Control and Prevention<br>Du, Huaidong; University of Oxford, Clinical Trial Service Unit & Epidemiological Studies Unit (CTSU), Nuffield Department of Population Health; University of Oxford, Medical Research Council Population Health Research Unit, Nuffield Department of Population Health<br>Yu, Min; Zhejiang Provincial Center for Diseases Control and Prevention, Department of NCDs Control and Prevention

Keywords:	Screen time, Behaviours, Lifestyle, Adolescents, Factors

SCHOLARONE™
Manuscripts

Prevalence of high screen time and associated factors among Chinese adolescents

Hao Wang[1], Jieming Zhong[1], Ruying Hu[1], Huaidong Du[2, 3], Min Yu[1]

Author affilitations

1. Department of NCDs Control and Prevention, Zhejiang Provincial Center for Diseases Control and Prevention, Hangzhou, China

2. Clinical Trial Service Unit & Epidemiological Studies Unit (CTSU), Nuffield Department of Population Health, University of Oxford, Oxford, UK

3. Medical Research Council Population Health Research Unit, Nuffield Department of Population Health, University of Oxford, Oxford, UK

Correspondence to Min Yu; myu@cdc.zj.cn

For peer review only - http://bmjopen.bmj.com/site/about/guidelines.xhtml

Strengths and limitations of this study

- This was a school-based study with relatively large sample size, high response rate and representative provincial samples from China.
- The study questionnaire involved a great number of behavioral risk factors, which might provide evidence for decision-maker to formulate intervention measures.
- The study was based on a cross-sectional study, which did not allow us establish the temporal relationship between factors and high ST.
- More detailed information about ST (such as duration of watching TV, playing computer and playing electronic games) did not covered in the questionnaire, which did not allow us to do further data analysis.

For peer review only

For peer review only - http://bmjopen.bmj.com/site/about/guidelines.xhtml

Abstract

Objective: To investigate the prevalence and correlates of high screen time (ST) among adolescents in Zhejiang, China.

Methods: A school-based cross-sectional survey was performed between April and May 2017. A total of 23 543 adolescents in grades 7-12 from 442 different schools were surveyed using anonymous self-administered questionnaires. Multivariate logistic regression models were used for data analyses.

Results: The mean age of the students was 15.6 years and 49.7% of them were girls. The prevalence of high ST (screen viewing ≥2 hours per day) was 42.4% (95%CI: 40.2-44.5), higher in boys than in girls (45.4% (95%CI: 42.8-48.0) vs. 39.1% (95%CI: 36.6-41.7)). No statistical significance of high ST prevalence was found between urban and rural areas (43.0% (95%CI: 37.2-48.7) vs. 42.1% (95%CI: 39.6-44.6)). The prevalence of high ST among middle school, academic high school, and vocational high school students were 35.3%, 30.0% and 73.5%, respectively. Multivariate logistic analysis showed that older age, students of vocational school, non-intact family, bad academic performance, bad self-reported health status, loneliness, consuming fruit <1 times or ≥3 times per day, and drinking carbonated beverages ≥3 times per day were positively associated with high ST, and students of academic high school, higher parental education, being physically active were negatively associated with high ST.

Conclusions: High ST is prevalent among adolescents and associated with a cluster of unhealthy behavioural risk factors in Zhejiang China.

Keywords: Screen time; Behaviours; Lifestyle; Adolescents; Factors

For peer review only - http://bmjopen.bmj.com/site/about/guidelines.xhtml

Introduction

With the rapid economic development, lifestyles of Chinese have been experiencing dramatic transition during the past decades. Increasing sedentary time and decreasing physical activity are becoming more and more obvious in China[1]. Meanwhile, prevalence of obesity and diabetes increased sharply[2 3]. Exposure to electronic screen products is known to be the most popular sedentary leisure activity among adolescents[4].

According to the report released by China Internet Information Center[5], the number of Internet users increased from 0.54 billion in 2012 increased to 0.71 billion in 2017. Meanwhile, the proportion of using mobile Internet increased from 72.2% in 2012 to 92.5% in 2017. Compared with the traditional desktops, smartphone was the easier for adolescents accessing Internet, which means more time will be spent on electronic screen products. Besides, in the United States, while the prevalence of US high school students who exceeded the recommended 2 or fewer hours/day of television viewing time decreased significantly from 43% in 1999 to 32% in 2013, the prevalence of students who spent more than 2 hours/day playing video or computer games nearly doubled from 22% to 41% in 2013[6]. It can be supposed that this transition will also happen in the future in China. Of all of the Chinese netizen, adolescents aged 10-19 years old accounted for approximately 20%[5].

In China, approximately 60% of inner-city adolescents had one or more screen products in their bedroom[7]. High screen time (ST) among adolescents has been increasingly recognized as a serious public health concern. High ST can be tracked from adolescents to early adulthood[8]. Previous studies indicated that high ST was not only associated with chronic diseases, such as obesity, metabolic syndrome etc[9-12], but also linked with psychological health of adolescents[13]. However, the evidence of high ST correlates mainly came from the western developed countries[14-16], with little known about the epidemiological characteristics and correlates in China. A previous Chinese study surveyed a total of 5 003 in-school adolescents and found 26% were exposed to ST for more than 2 h/day[17]. However, this study only recruited middle school students, but did not cover high school students. Besides, it was in 2010 year when the survey was implemented. During the past seven years, technology of screen products developed rapidly and screen products spread explosively worldwide, which make adolescents easier to access these products. Therefore, the objective of this study was to examine the prevalence of high ST and its correlate factors among adolescents in Zhejiang Province, China.

Methods

Survey design

A cross-sectional study was carried out between April and May 2017 in Zhejiang province, China. The survey was a two-stage sampling design. In stage one, 30 counties, including 12 urban areas and 18 rural areas, were sampled from all 90 counties in Zhejiang. In stage two, 10

For peer review only - http://bmjopen.bmj.com/site/about/guidelines.xhtml

classes of middle school, 5 classes of academic high school, and 5 classes of vocational high school were selected randomly within each chosen counties, respectively. All the students in the chosen classes were invited to participate in the survey. The survey questionnaire was developed based on Youth Risk Behaviour Survey conducted by the Centers for Disease Control and Prevention[18] and the international Global School-based Student Health Survey supported by the World Health Organization[19]. Questionnaire covered demographic characteristics, tobacco and alcohol use, physical activity, dietary habit, violence, injury, sexual behaviours. Subjects filled in the anonymous self-administrated questionnaire in the classrooms. The finished questionnaires were handed in on the spot.

Measures

Participants' ST was assessed through the question: "On an average school day, how many hours do you watch television (TV), play pad or electronic games or use a smartphone or computer for something that is not school work? (Answer options: I do not watch TV, play pad or electronic games or use a smartphone or computer for something that is not school work, <1h/d, 1h/d, 2h/d, 3h/d, 4h/d, ≥5h/d).”; Participants were considered as high screen-time users if they answered had watched screen more than 2 hours on an average school day[20 21]. Being physically active was defined as a total of at least 60 minutes physical activity every day among adolescents[22]. Other variables were described in Table 1.

Quality Control

Local Center for Disease Control and Prevention took charge of the survey. All of the surveyors were trained before the survey. In order to improve response rate, every recruited student was given a pencil box as a gift. The survey was anonymous, and without filling in the participants' name on the questionnaire.

Ethics Statement

This study was approved by the ethics committee of Zhejiang Provincial Centre for Disease Control and Prevention. Written informed consent was given before survey, and obtained from all participants and their guardians. The ethics committee approved this procedure.

Statistical analysis

A weighting factor was applied to each student record to adjust for non-response and for the varying probabilities of selection. The weight used for estimation in this survey is given by: W= W1 * W2 * f1* f2. W1 = the inverse of the probability of selecting the county; W2= the inverse of the probability of selecting the classroom within the county. f1= a student-level nonresponse adjustment factor calculated by class. f2= a post-stratification adjustment factor calculated by grade[23].

For peer review only

For peer review only - http://bmjopen.bmj.com/site/about/guidelines.xhtml

Continuous variables were shown as the mean ± standard deviation. The prevalence of high ST was given as percent and 95% confidence intervals (CI). Categorical variables between groups were performed using Chi-square test. Weighted prevalence between groups was calculated with Rao-Scott Chi-square test. Multivariate logistic regression was used to ascertain factors related with high ST. All analyses were performed with SAS software V.9.3. All statistical tests were two tailed, and *P*-values <0.05 were considered statistically significant.

Results

Descriptive statistics

A total of 24157 students were selected from grades 7-12 in 442 different schools. Due to missing or incompletion of questionnaires and refusal to participation, 614 students were excluded, yielding 23,543 eligible participants (response rate 97.5%) in the final analysis. Of which, 12 068 (51.3%) were boys and the average age was 15.6 years for both boys and girls. 12 207 (51.9%) of the students were from middle schools, 6 477 (27.5%) were from academic high schools and 4 859 (20.6%) came from vocational high school.

Table 2 presented that 37.7% boys and 39.0% girls came from urban areas, respectively. Boys were more likely to describe personal health status as very good or good than girls (56.0% vs.49.3%). The proportion of boys who never or occasionally felt lonely was higher than that of girls (67.0% vs.61.3%). Nearly 16.7% students reported to be physically active every day. Approximately 70.6% students reported consuming breakfast every day. The percentages of adolescents consuming fruit and vegetable <1 times per day were 28.7% and 8.0%, respectively. There was no sex difference in the frequency of carbonated drinks consumption (*P*=0.19).

The prevalence of high ST

Just as shown in the Table 3, the prevalence of high ST was 42.4% (95%CI: 40.2-44.5), higher in boys than in girls (45.4% vs. 39.1%). There was no statistical significance of high ST prevalence between urban and rural areas (43.0% vs. 42.1%). With the increase of age, the prevalence of high ST increased gradually (*P*<0.0001). The prevalence of high ST among middle school, academic high school, and vocational high school was 35.3%, 30.0% and 73.5%, respectively.

Logistic regression analysis

Table 4 demonstrated that univariate and multivariate logistic regression analysis result of socio-demographic and health-related behaviors of high ST. Univariate analysis showed that age, types of school, parental marital status, parental education level, academic performance, self-reported health status, loneliness, being physically active, having breakfast, fruit consumption, vegetable consumption, drinking carbonated beverages were significantly associated with high ST.

For peer review only

After adjusted all other variables in the model, multivariate analysis showed that students living in non-intact families had a 1.3 times higher risk of high ST (OR=1.26, 95%CI: 1.13-1.41). Compared to students whose paternal education level was primary or below, students whose paternal education were middle or high school and college or above had 0.9 times and 0.7 times less probability of high ST. In contrast with students whose maternal education level was primary or below, those whose maternal education level was college or above had 0.7 times less likelihood of high ST(OR=0.66, 95%CI: 0.58-0.76). Students with bad academic performance were 2.1 times more likely to expose to high ST than those with excellent academic performance (OR=2.07, 95%CI: 1.86-2.30). Students who often or always felt lonely were 1.2 times more likely to expose to high ST than Students who never or occasionally felt lonely (OR=1.20, 95%CI: 1.08-1.34). Compared to students who were not physically active within the past 7 days, those being physically active 1-2 days had a lower risk of high ST (OR=0.90, 95%CI: 0.81-0.99). Compared to students consuming fruits 1-2 times per day, both students consuming fruits < 1 times per day and ≥ 3 times per day had higher risks of high ST((OR=1.44, 95%CI: 1.29-1.60), (OR=1.18, 95%CI: 1.01-1.38), respectively) Compared to students who never consume carbonated beverages, those consuming carbonated beverages ≥ 3 times per day had higher odds of high ST (OR=1.29, 95%CI: 1.03-1.60).

For peer review only - http://bmjopen.bmj.com/site/about/guidelines.xhtml

Discussion

Differences in the questions used to evaluate ST make it fail to compare directly our results with the results of previous studies. However, we found that more than two out of five students exceeded the recommended maximum ST of 2h/d, signifying excessive exposure to electronic screen products are becoming more and more common among adolescents in China. A study, using the similar questions as ours, conducted in Brazilian in 2013-2014, showed that 59.5% of adolescents aged 12-17 years were exposed to electronic screen ≥ 2h/d[24], higher than our study. Consistent with results from other studies[7 17 25], it was boys, rather than girls, had higher ST prevalence. This gender difference can be mainly explained by the fact that boys tend to be more attracted by computers games (such as sports, racing, fighting, shooting) than girls[26]. Another possible reason was that girls usually spend more time on homework than boys in China. Similar with other studies[25 27], the prevalence of high ST increased with the increase of age. Notably, among students of three different types of school, vocational high school had the highest prevalence of high ST, which means students of vocational high school should be target population of intervention. A possible explanation was that vocational high school students did not have to face competitive high school or college entrance examination, which is different from middle school and academic high school students. Hence, they had more time to spend on electronic screen products.

In our study, an inverse association existed between parental education level and high ST. It may be because highly-educated parents were inclined to make stricter rules of children's ST at homes as compared to low-educated parents. In China, approximately 50% of families had no relative rules of ST[7]. Having ST rules at homes may have protective effect on children's excessive ST[7]. One possible explanation was that bad health status might refrain students from engaging in physical activity and increase likelihood of excessive exposure to electronic screen products. Another possible explanation was that excessive exposure to electronic screen products damage adolescents' health. These are the possible reason why bad health status was positively associated with high ST in our study. Compared to those who never or occasionally feel lonely, those often or always feel lonely had much higher odds of high ST, which was in line with other study[28].

Importantly, our study indicated that being physically active were negatively associated with high ST. In 2007, Chinese government proposed the guideline about physical activity at least 60 minutes every day among adolescents[22]. Reducing ST and increasing physical activity can produce psychological benefits that can impact quality of life, academic performance and self-esteem[29 30]. In our study, nearly 16.7% students reported to being physical active every day, which was lower than average level in China (22.7%)[31], meaning physical activity among adolescents should be strengthened in Zhejiang. A previous study found that Chinese junior

For peer review only - http://bmjopen.bmj.com/site/about/guidelines.xhtml

students were lack of awareness on physical activity[32], and so the related intervention should be taken actively to improve the current situation. More students should be encouraged to engage in physical activity outdoors.

Relationship of ST with having breakfast differed according to age and race. Lipsky et al. analysed a representative sample of US adolescents and found that in participants younger than 13, there was a positive relationship between TV viewing and skipping breakfast in white youth, but an inverse relationship in black youth and no significant relationship in Hispanic youth. Meanwhile, skipping breakfast was not related to TV viewing in youth aged 13 or older[33], which were similar with our study. In China, after 6 years of primary education, adolescents usually enter middle school at 13 years of age for 3 years of middle education.

The dietary guidelines for Chinese, recommended 200-350 grams fruits and 300-500 grams vegetables should be consumed for an individual every day. It is impossible for us to get exact grams of fruits and vegetables through a self-administrated survey. Our study found over 70% of students consumed fruits ≥1 times per day, higher than the results of the United States adolescents (64%)[34]. A previous study found television viewing was inversely related to intake of fruit (OR=0.92) and vegetable (OR=0.95) among the United States adolescents[33]. In our study, compared to students consuming fruits 1-2 times per day, those consuming fruits <1 times per day had a higher likelihood of high ST. Interestingly, those consuming fruits ≥3 times per day also had a higher probability of high ST, different from Lipsky et al.' s study[33]. One possible explanation was fruit intake was only divided into two group ("≥1 times per day" group and "<1 times per day" group) in Lipsky et al.' s study, which fail to describe clearly the association between different frequency of fruit intake with ST. Another possible explanation was when excessive exposure to electronic screen occurs among adolescents at homes, parents seem to provide fruits for children to help them regain body strength. In the present study, half of the students consumed vegetable ≥3 times per day, higher than the United States (18.5%)[35]. Tough vegetable intake was negatively associated with high ST in univariate logistic regression, statistical significance disappeared in multivariate analysis.

Many studies have demonstrated that sugar sweetened beverages (SSB) were linked with chronic diseases (e.g., obesity, diabetes, hypertension, stroke)[36-39]. Schulze et al. found that those consuming ≥1 SSB per day had an 83% greater risk of developing type 2 diabetes compare to those consuming < 1 SSB per month[36]. In our study, 6.9% of students reported consuming carbonated soft drinks ≥1 time per day. Though the percentage was far lower than in the United States (20.4%)[40], it should raise concern about SSB consumption among adolescents in China. Gebremariam et al. found that an increase in TV viewing by an hour per day was associated with the consumption of 30 ml per day more soft drinks in Greece and 90 ml per day more soft drinks in Switzerland[14]. The present study showed that students consuming

For peer review only - http://bmjopen.bmj.com/site/about/guidelines.xhtml

carbonated drinks ≥ 3 times per day have a 1.3 times higher risk of high ST, which was in correspondence with findings from a previous study[41]. The possible explanation was that watching television was the most prevalent ST behaviours among Chinese adolescents[7]. The majority of food advertisements were for unhealthy food[42]. Carbonated drinks advertisements on television (e.g., Coca-Cola, Pepsi, and Sprite) stimulated the consumption of carbonated drinks.

With the development of internet technology and emergence of new screen products, it is inevitable for adolescents to get more chances for electronic screen products. Hence, comprehensive measures should be taken, including enhancing public awareness of hazards on excessive ST, increasing physical activity among adolescents, reducing frequency of unhealthy food broadcast on television, cultivating healthy dietary lifestyles among adolescents, for reducing the prevalent High ST in China.

There are several limitations. First, the study was based on a cross-sectional study, which did not allow us establish the temporal relationship between factors and ST. Second, more detailed information about ST (such as duration of watching TV, playing computer and playing electronic games) did not covered in the questionnaire, which did not allow us to do further data analysis. Third, all of the data was self-reported by students, and without measurement, which may result in information bias. Fourth, we only collected the information of ST on school days, and did not covered non-school days. Students usually spent more ST on non-school days than on school days[7], which may yield an underestimation of ST among adolescents.

Overall, our study extended the current literature by describing the patterns and associations of high ST among representative provincial sample in China, and found that high ST is prevalent among adolescents and associated with a cluster of unhealthy behavioural risk factors in Zhejiang China.

Acknowledgements The authors would like to thank all the students, parents, teachers and local officials for their participation, assistance and co-operation.

Contributor HW designed the study, and collected and analyzed the data with MY. JZ and RH were involved in data interpretation. HD took part in data analysis and revised the manuscript. All the authors have read and approved the final submitted version.

Competing interests None declared.

Funding The work was supported by grant (2016YFC0900502) from National Key Research and Development Program of China.

Ethics approval Ethics Committee of Zhejiang Provincial Centre for Disease Control and Prevention.

Data sharing statement No additional data are available

For peer review only - http://bmjopen.bmj.com/site/about/guidelines.xhtml

References

1. Ng SW, Howard AG, Wang HJ, et al. The physical activity transition among adults in China: 1991-2011. *Obes Rev* 2014;15 Suppl 1:27-36. doi: 10.1111/obr.12127
2. Ji CY, Working Group on Obesity in C. Report on childhood obesity in China (4) prevalence and trends of overweight and obesity in Chinese urban school-age children and adolescents, 1985-2000. *Biomed Environ Sci* 2007;20(1):1-10.
3. Wang L, Gao P, Zhang M, et al. Prevalence and Ethnic Pattern of Diabetes and Prediabetes in China in 2013. *JAMA* 2017;317(24):2515-23. doi: 10.1001/jama.2017.7596
4. Trang NH, Hong TK, Dibley MJ. Cohort profile: Ho Chi Minh City Youth Cohort--changes in diet, physical activity, sedentary behaviour and relationship with overweight/obesity in adolescents. *BMJ Open* 2012;2(1):e000362. doi: 10.1136/bmjopen-2011-000362
5. Centre CINI. China Internet Development Statistics Report. Beijing, 2016.
6. Kann L, Kinchen S, Shanklin SL, et al. Youth risk behavior surveillance--United States, 2013. *MMWR Suppl* 2014;63(4):1-168.
7. Jiang XX, Hardy LL, Ding D, et al. Recreational screen-time among Chinese adolescents: a cross-sectional study. *J Epidemiol* 2014;24(5):397-403.
8. Busschaert C, Cardon G, Van Cauwenberg J, et al. Tracking and predictors of screen time from early adolescence to early adulthood: a 10-year follow-up study. *J Adolesc Health* 2015;56(4):440-8. doi: 10.1016/j.jadohealth.2014.11.016
9. Grontved A, Ried-Larsen M, Moller NC, et al. Youth screen-time behaviour is associated with cardiovascular risk in young adulthood: the European Youth Heart Study. *Eur J Prev Cardiol* 2014;21(1):49-56. doi: 10.1177/2047487312454760
10. Kang HT, Lee HR, Shim JY, et al. Association between screen time and metabolic syndrome in children and adolescents in Korea: the 2005 Korean National Health and Nutrition Examination Survey. *Diabetes Res Clin Pract* 2010;89(1):72-8. doi: 10.1016/j.diabres.2010.02.016
11. Robinson TN, Banda JA, Hale L, et al. Screen Media Exposure and Obesity in Children and Adolescents. *Pediatrics* 2017;140(Suppl 2):S97-S101. doi: 10.1542/peds.2016-1758K
12. Mark AE, Janssen I. Relationship between screen time and metabolic syndrome in adolescents. *J Public Health (Oxf)* 2008;30(2):153-60. doi: 10.1093/pubmed/fdn022
13. Page AS, Cooper AR, Griew P, et al. Children's screen viewing is related to psychological difficulties irrespective of physical activity. *Pediatrics* 2010;126(5):e1011-7. doi: 10.1542/peds.2010-1154
14. Gebremariam MK, Chinapaw MJ, Bringolf-Isler B, et al. Screen-based sedentary time: Association with soft drink consumption and the moderating effect of parental education in European children: The ENERGY study. *PLoS One* 2017;12(2):e0171537. doi: 10.1371/journal.pone.0171537
15. Lee EY, Hunter S, Leatherdale ST, et al. Sociodemographic correlates of physical activity and screen time among adolescents in Canada and Guatemala: Results from the COMPASS system. *Glob Health Promot* 2017:1757975917710804. doi: 10.1177/1757975917710804
16. Brunetti VC, O'Loughlin EK, O'Loughlin J, et al. Screen and nonscreen sedentary behavior and sleep in adolescents. *Sleep Health* 2016;2(4):335-40. doi: 10.1016/j.sleh.2016.09.004
17. Cao H, Qian Q, Weng T, et al. Screen time, physical activity and mental health among urban adolescents in China. *Prev Med* 2011;53(4-5):316-20. doi: 10.1016/j.ypmed.2011.09.002
18. Centers for Disease Control and Prevention . 2017 State and Local Youth Risk Behavior Survey; 2017 [Available from: https://www.cdc.gov/healthyyouth/data/yrbs/questionnaires.htm accessed 21/11 2017.
19. World Health Organization.Global school-based students health survey(GSHS) 2015 [updated 7/6. Available from: http://www.who.int/ncds/surveillance/gshs/nepal/en/ accessed 21/11 2017.

For peer review only - http://bmjopen.bmj.com/site/about/guidelines.xhtml

20. American Academy of Pediatrics. Committee on Public E. American Academy of Pediatrics: Children, adolescents, and television. *Pediatrics* 2001;107(2):423-6.
21. Barlow SE, Expert C. Expert committee recommendations regarding the prevention, assessment, and treatment of child and adolescent overweight and obesity: summary report. *Pediatrics* 2007;120 Suppl 4:S164-92. doi: 10.1542/peds.2007-2329C
22. China National Education. Suggestion of CPC Central Committee and State Council on strengthening the youth sports to enhance youth physical. *Chin J Sch Health* 2007;28(6):481-83.
23. Pengpid S, Peltzer K. Alcohol use and associated factors among adolescent students in Thailand. *West Indian Med J* 2012;61(9):890-6.
24. Cureau FV, Ekelund U, Bloch KV, et al. Does body mass index modify the association between physical activity and screen time with cardiometabolic risk factors in adolescents? Findings from a country-wide survey. *Int J Obes (Lond)* 2017;41(4):551-59. doi: 10.1038/ijo.2016.210
25. Nguyen PV, Hong TK, Nguyen DT, et al. Excessive screen viewing time by adolescents and body fatness in a developing country: Vietnam. *Asia Pac J Clin Nutr* 2016;25(1):174-83. doi: 10.6133/apjcn.2016.25.1.21
26. Hartmann T, Klimmt C. Gender and Computer Games: Exploring Females' Dislikes. *Journal of Computer-Mediated Communication* 2006;11(4): 910–31 doi: 10.1111/j.1083-6101.2006.00301.x
27. Hoyos Cillero I, Jago R, Sebire S. Individual and social predictors of screen-viewing among Spanish school children. *Eur J Pediatr* 2011;170(1):93-102. doi: 10.1007/s00431-010-1276-6
28. Wu X, Tao S, Zhang Y, et al. Low physical activity and high screen time can increase the risks of mental health problems and poor sleep quality among Chinese college students. *PLoS One* 2015;10(3):e0119607. doi: 10.1371/journal.pone.0119607
29. Suchert V, Hanewinkel R, Isensee B. Sedentary behavior and indicators of mental health in school-aged children and adolescents: A systematic review. *Prev Med* 2015;76:48-57. doi: 10.1016/j.ypmed.2015.03.026
30. Tremblay MS, Leblanc AG, Janssen I, et al. Canadian sedentary behaviour guidelines for children and youth. *Appl Physiol Nutr Metab* 2011;36(1):59-64; 65-71. doi: 10.1139/H11-012
31. Zhang X, Song Y, Yang TB, et al. [Analysis of current situation of physical activity and influencing factors in Chinese primary and middle school students in 2010]. *Zhonghua Yu Fang Yi Xue Za Zhi* 2012;46(9):781-8.
32. Ren YJ, Liu QM, Lu J, et al. [Behavior and knowledge on physical activity among urban junior students in Hangzhou]. *Zhonghua Liu Xing Bing Xue Za Zhi* 2012;33(7):672-5.
33. Lipsky LM, Iannotti RJ. Associations of television viewing with eating behaviors in the 2009 Health Behaviour in School-aged Children Study. *Arch Pediatr Adolesc Med* 2012;166(5):465-72. doi: 10.1001/archpediatrics.2011.1407
34. Eaton DK, Kann L, Kinchen S, et al. Youth risk behavior surveillance - United States, 2011. *MMWR Surveill Summ* 2012;61(4):1-162.
35. Lowry R, Michael S, Demissie Z, et al. Associations of Physical Activity and Sedentary Behaviors with Dietary Behaviors among US High School Students. *J Obes* 2015;2015:876524. doi: 10.1155/2015/876524
36. Schulze MB, Manson JE, Ludwig DS, et al. Sugar-sweetened beverages, weight gain, and incidence of type 2 diabetes in young and middle-aged women. *JAMA* 2004;292(8):927-34. doi: 10.1001/jama.292.8.927
37. Palmer JR, Boggs DA, Krishnan S, et al. Sugar-sweetened beverages and incidence of type 2 diabetes mellitus in African American women. *Arch Intern Med* 2008;168(14):1487-92. doi: 10.1001/archinte.168.14.1487
38. Dhingra R, Sullivan L, Jacques PF, et al. Soft drink consumption and risk of developing cardiometabolic risk factors and the metabolic syndrome in middle-aged adults in the community. *Circulation* 2007;116(5):480-8. doi: 10.1161/CIRCULATIONAHA.107.689935

For peer review only - http://bmjopen.bmj.com/site/about/guidelines.xhtml

39. Fung TT, Malik V, Rexrode KM, et al. Sweetened beverage consumption and risk of coronary heart disease in women. *Am J Clin Nutr* 2009;89(4):1037-42. doi: 10.3945/ajcn.2008.27140
40. Kann L, McManus T, Harris WA, et al. Youth Risk Behavior Surveillance - United States, 2015. *MMWR Surveill Summ* 2016;65(6):1-174. doi: 10.15585/mmwr.ss6506a1
41. Kelishadi R, Mozafarian N, Qorbani M, et al. Association between screen time and snack consumption in children and adolescents: The CASPIAN-IV study. *J Pediatr Endocrinol Metab* 2017;30(2):211-19. doi: 10.1515/jpem-2016-0312
42. Li D, Wang T, Cheng Y, et al. The extent and nature of television food advertising to children in Xi'an, China. *BMC Public Health* 2016;16(1):770. doi: 10.1186/s12889-016-3468-0

For peer review only

For peer review only - http://bmjopen.bmj.com/site/about/guidelines.xhtml

Table 1 questions comprising variables included in the survey

Variables	Questions and options
Parental education level	What is the highest level of education your father/mother has obtained? (Answer options: primary school or below, middle school, high school, college or university, master graduates or above, unknown)（separately for father and mother）
Parental marital status	What is your parent current marital status? (Answer options: married, divorced, widowed, separated)
Academic performance	How would you describe your grades in your class? (Answer options: Excellent, middle, bad)
Self-report health	In general, how would you describe your health status? (Answer options: very good, good, fair, bad, very bad)
Health status	In general, which do you think is your health? (Answer options: very good, good, fair, poor, very poor, unknown)
Loneliness	During the past 12 months, did you ever feel lonely? (Answer options: never, occasional, sometimes, often, always)
Physical activity	During the past 7 days, on how many days were you physically active for a total of at least 60 minutes per day?(Answer options: none, 1 days, 2 days, 3 days, 4 days, 5 days, 6 days, 7 days)
Breakfast	During the past 7 days, on how many days did you eat breakfast? (Answer options: 0 days, 1 days, 2 days, 3 days, 4 days, 5 days, 6 days, 7 days)
Fruit	During the past 30 days, how many times per day did you **usually** eat fruit, such as apples, oranges, mangoes, or papayas? (Answer options: none, < 1 h/d, 1 time /d, 2 times/d, 3 times/d, 4 times /d, ⩾ 5 times/d)
Vegetable	During the past 30 days, how many times per day did you **usually** eat vegetables, such as cauliflower, cabbage? (Answer options: none, < 1 h/d, 1 time /d, 2 times/d, 3 times/d, 4 times /d, ⩾ 5 times/d)
Carbonated soft drinks	During the past 30 days, how many times per day did you **usually** drink carbonated soft drinks, such as Coca-Cola, Pepsi, or Sprite? (Do not include diet soft drinks.) (Answer options: none, < 1 times/d, 1 time /d, 2 times/d, 3 times/d, 4 times /d, ⩾ 5 times/d)

For peer review only - http://bmjopen.bmj.com/site/about/guidelines.xhtml

Table 2 socio-demographic and characteristics of adolescents participating in the survey from Zhejiang (N=23543)

Characteristics	Total	Boys	Girls	Chi-square	*P*
Age range (years)				1.98	0.577
≤13	5159(21.9)	2689(22.3)	2470(21.5)		
14	4300(18.3)	2192(18.1)	2108(18.4)		
15	3730(15.8)	1905(15.8)	1825(15.9)		
≥16	10354(44.0)	5282(43.8)	5072(44.2)		
Area				4.68	0.031
Urban	9022(38.3)	4544(37.7)	4478(39.0)		
Rural	14521(61.7)	7524(62.3)	6997(61.0)		
Types of school				9.63	0.008
Middle school	12207(51.8)	6364(52.7)	5843(50.9)		
Academic high school	6477(27.5)	3223(26.7)	3254(28.4)		
Vocational high school	4859(20.6)	2481(20.6)	2378(20.7)		
Parental marital status				12.56	0.0004
Married	21151(89.8)	10924(90.5)	10227(89.1)		
Others	2392(10.2)	1144(9.5)	1248(10.9)		
Paternal education				10.68	0.0136
Primary or below	13568(57.6)	6908(57.2)	6660(58.0)		
Middle or high school	5100(21.7)	2628(21.8)	2472(21.5)		
College or above	3129(13.3)	1575(13.1)	1554(13.5)		
Unknown	1746(7.4)	957(7.9)	789(7.0)		
Maternal education				44.34	<.0001
Primary or below	14530(61.7)	7292(60.4)	7238(63.1)		
Middle or high school	4363(18.5)	2271(18.8)	2092(18.2)		
College or above	2736(11.6)	1392(11.5)	1344(11.7)		
Unknown	1914(8.1)	1113(9.2)	801(7.0)		
Academic performance				118.53	<.0001
Excellent	5448(23.1)	2731(22.6)	2717(23.7)		
Middle	11765(50.0)	5727(47.5)	6038(52.6)		
Bad	6330(26.9)	3610(29.9)	2720(23.7)		
Self-reported health				129.52	<.0001
Very good/good	12415(52.7)	6758(56.0)	5657(49.3)		
Fair	9563(40.6)	4495(37.2)	5068(44.2)		
Bad/very bad	1293(5.5)	650(5.4)	643(5.6)		
Unknown	272(1.2)	165(1.4)	107(0.9)		
Loneliness				84.27	<.0001
Never/Occasionally	15122(64.2)	8082(67.0)	7040(61.3)		
Sometimes	5783(24.6)	2698(22.4)	3085(26.9)		
Often/Always	2638(11.2)	1288(10.6)	1350(11.8)		
Being physically active (d/wk)				411.53	<.0001
0	4883(20.7)	2079(17.2)	2804(24.5)		
1-2	5690(24.2)	2703(22.4)	2987(26.0)		
3-5	8050(34.2)	4237(35.1)	3813(33.2)		
6-7	4920(20.9)	3049(25.3)	1871(16.3)		
Breakfast(d/wk)				21.95	<.0001

For peer review only - http://bmjopen.bmj.com/site/about/guidelines.xhtml

0	473(2.0)	289(2.39)	184(1.60)		
1-2	599(2.5)	298(2.47)	301(2.62)		
3-4	1249(5.3)	667(5.53)	582(5.07)		
≥5	21222(90.2)	10814(89.61)	10408(90.70)		
Fruit(times/d)				9.26	0.01
≥3	6847(29.1)	3453(28.6)	3394(29.6)		
1-2	9945(42.2)	5213(43.2)	4732(41.2)		
<1	6751(28.7)	3402(28.2)	3349(29.2)		
Vegetable(times/d)				1.83	0.40
≥3	11775(50.0)	5984(49.6)	5791(50.5)		
1-2	9884(42.0)	5108(42.3)	4776(41.6)		
<1	1884(8.0)	976(8.1)	908(7.9)		
Carbonated drinks				4.72	0.19
never	9133(38.8)	4717(39.1)	4416(38.5)		
1-6 times/wk	12792(54.3)	6529(54.1)	6263(54.6)		
1-2 times/d	1146(4.9)	600(5.0)	546(4.7)		
≥3 times/d	472(2.0)	222(1.8)	250(2.2)		

For peer review only

For peer review only - http://bmjopen.bmj.com/site/about/guidelines.xhtml

Table 3 Weighted prevalence of high ST among adolescents in China by different characteristics

Characteristics	Prevalence (%)*	Rao-Scott Chi-Square	*P*
Sex		18.03	<.0001
Boys	45.4(42.8-48.0)		
Girls	39.1(36.6-41.7)		
Areas		0.06	0.81
Urban	43.0(37.2-48.7)		
Rural	42.1(39.6-44.6)		
Age group(y)		89.05	<.0001
<14	31.0(27.4-34.7)		
14-15	36.4(33.6-39.3)		
15-16	43.5(40.2-46.9)		
≥16	49.6(46.0-53.1)		
Types of school		404.57	<.0001
Middle school	35.3(33.0-37.6)		
Academic high school	30.0(27.2-32.9)		
Vocational high school	73.5(69.5-77.5)		

*Based on the weighted data.

For peer review only

For peer review only - http://bmjopen.bmj.com/site/about/guidelines.xhtml

Table 4 crude and adjusted odd ratio of factors associated with high ST among adolescents from Zhejiang

Characteristics	COR(95%CI)	AOR(95%CI)a
Age groups(ref: ≤13 years)		
14 years	**1.27(1.06-1.54)** #	**1.24(1.05-1.46)** #
15 years	**1.71(1.38-2.13)** &	**1.41(1.16-1.71)** $
≥16 years	**2.18(1.76-2.72)** &	**1.34(1.07-1.69)** *
Rural(ref: urban)	0.97(0.73-1.27)	0.99(0.85-1.15)
Types of school (ref: middle school)		
Academic high school	**0.79(0.67-0.93)** *	**0.61(0.49-0.77)** &
Vocational high school	**5.09(4.05-6.40)** &	**3.71(2.83-4.85)** &
Parental marital status(ref: married)		
Others	**1.52(1.37-1.69)** &	**1.26(1.13-1.41)** &
Paternal education (ref: primary or below)		
Middle or high school	**0.75(0.68-0.82)** &	**0.88(0.81-0.98)** *
College or above	**0.40(0.33-0.48**) &	**0.69(0.58-0.82**) &
Unknown	0.98(0.85-1.13)	0.83(0.68-1.01)
Maternal education (ref: primary or below)		
Middle or high school	**0.75(0.67-0.83)** &	0.90(0.82-1.00)
College or above	**0.38(0.33-0.45)** &	**0.66(0.58-0.76)** &
Unknown	**1.14(1.00-1.30)** #	1.18(0.99-1.41)
Academic performance(ref: excellent)		
Middle	**1.55(1.42-1.69)** &	**1.36(1.25-1.48)** &
Bad	**2.35(2.12-2.60)** &	**2.07(1.86-2.30)** &
Self-reported health (ref: very good/good)		
Fair	**1.39(1.30-1.49)** &	**1.14(1.07-1.22)** &
Bad/very bad	**1.59(1.38-1.84)** &	**1.31(1.14-1.49)** &
Unknown	**1.46(1.07-2.00)** #	1.11(0.78-1.57)
Loneliness(ref: never/occasionally)		
Sometimes	**1.30(1.20-1.40)** &	**1.20(1.09-1.32)** &
Often/Always	**1.38(1.24-1.54)** &	**1.20(1.08-1.34)** $

For peer review only - http://bmjopen.bmj.com/site/about/guidelines.xhtml

Being physically active (ref: 0 d/wk)		
1-2 d/wk	**0.81(0.74-0.89)** &	**0.90(0.81-0.99)#**
3-5 d/wk	**0.74(0.68-0.82)** &	**0.91(0.83-0.99)** #
6-7 d/wk	**0.69(0.61-0.79)** &	0.93(0.82-1.05)
Breakfast (ref: 0 d/wk)		
1-2 d/wk	**1.38(1.03-1.85)** #	1.25(0.92-1.70)
3-4 d/wk	1.21(0.97-1.51)	1.04(0.81-1.33)
≥5 d/wk	**0.70(0.57-0.86)** $	0.95(0.74-1.22)
Fruit (ref: 1-2 times/d)		
<1 times/d	**2.36(2.09-2.66)** &	**1.44(1.29-1.60)**&
≥3 times/d	**2.01(1.65-2.44)** &	**1.18(1.01-1.38)** #
Vegetable(ref: 1-2 times/d)		
<1 times/d	**1.28(1.13-1.45)** &	0.99(0.86-1.14)
≥3 times/d	**1.27(1.16-1.39) &**	0.98(0.90-1.07)
Carbonated drinks (ref: never)		
1-6 times/wk	1.02(0.95-1.09)	1.02(0.96-1.09)
1-2 times/d	0.98(0.83-1.15)	0.99(0.84-1.18)
≥3 times/d	**1.50(1.25-1.80)** &	**1.29(1.03-1.60)** #

Bold numbers represent significant results

COR: Crude odds ratio. AOR: Adjusted odds ratios. CI: confidence intervals. #: P<0.05. *: P<0.01. $: P<0.001. & P<0.0001

a Adjusted for all other covariates listed in the table.

For peer review only

STROBE Statement—checklist of items that should be included in reports of observational studies

	Item No	Recommendation
**Title and abstract**	1 **YES Title:page1 Abstract: page3**	(*a*) Indicate the study's design with a commonly used term in the title or the abstract
		(*b*) Provide in the abstract an informative and balanced summary of what was done and what was found
**Introduction**		
Background/rationale	2 **YES Page4**	Explain the scientific background and rationale for the investigation being reported
Objectives	3 **YES Page 3**	State specific objectives, including any prespecified hypotheses
**Methods**		
Study design	4 **YES Page4-5**	Present key elements of study design early in the paper
Setting	5 **YES Page4-5**	Describe the setting, locations, and relevant dates, including periods of recruitment, exposure, follow-up, and data collection
Participants	6 **YES Page4-5**	(*a*) *Cohort study*—Give the eligibility criteria, and the sources and methods of selection of participants. Describe methods of follow-up *Case-control study*—Give the eligibility criteria, and the sources and methods of case ascertainment and control selection. Give the rationale for the choice of cases and controls *Cross-sectional study*—Give the eligibility criteria, and the sources and methods of selection of participants
		(*b*) *Cohort study*—For matched studies, give matching criteria and number of exposed and unexposed *Case-control study*—For matched studies, give matching criteria and the number of controls per case
Variables	7 **YES Page15**	Clearly define all outcomes, exposures, predictors, potential confounders, and effect modifiers. Give diagnostic criteria, if applicable
Data sources/ measurement	8* **YES Page 5, 15**	For each variable of interest, give sources of data and details of methods of assessment (measurement). Describe comparability of assessment methods if there is more than one group
Bias	9 **YES Page 5**	Describe any efforts to address potential sources of bias
Study size	10 **YES Page4-5**	Explain how the study size was arrived at
Quantitative variables	11 **YES**	Explain how quantitative variables were handled in the analyses. If applicable, describe which groupings were chosen and why
Statistical methods	12 **YES Page 5**	(*a*) Describe all statistical methods, including those used to control for confounding
		(*b*) Describe any methods used to examine subgroups and interactions
		(*c*) Explain how missing data were addressed
		(*d*) *Cohort study*—If applicable, explain how loss to follow-up was addressed *Case-control study*—If applicable, explain how matching of cases and

For peer review only - http://bmjopen.bmj.com/site/about/guidelines.xhtml

		controls was addressed<br>*Cross-sectional study*—If applicable, describe analytical methods taking account of sampling strategy
		(*e*) Describe any sensitivity analyses
**Results**		
Participants	13* **YES Page 6**	(a) Report numbers of individuals at each stage of study—eg numbers potentially eligible, examined for eligibility, confirmed eligible, included in the study, completing follow-up, and analysed
		(b) Give reasons for non-participation at each stage
		(c) Consider use of a flow diagram
Descriptive data	14* **YES Page 16-17**	(a) Give characteristics of study participants (eg demographic, clinical, social) and information on exposures and potential confounders
		(b) Indicate number of participants with missing data for each variable of interest
		(c) *Cohort study*—Summarise follow-up time (eg, average and total amount)
Outcome data	15* **YES Page 6 16-17**	*Cohort study*—Report numbers of outcome events or summary measures over time
		Case-control study—Report numbers in each exposure category, or summary measures of exposure
		Cross-sectional study—Report numbers of outcome events or summary measures
Main results	16 **YES Page18-20**	(*a*) Give unadjusted estimates and, if applicable, confounder-adjusted estimates and their precision (eg, 95% confidence interval). Make clear which confounders were adjusted for and why they were included
		(*b*) Report category boundaries when continuous variables were categorized
		(*c*) If relevant, consider translating estimates of relative risk into absolute risk for a meaningful time period
Other analyses	17 **No**	Report other analyses done—eg analyses of subgroups and interactions, and sensitivity analyses
**Discussion**		
Key results	18 **YES Page 8-10**	Summarise key results with reference to study objectives
Limitations	19 **YES Page 10**	Discuss limitations of the study, taking into account sources of potential bias or imprecision. Discuss both direction and magnitude of any potential bias
Interpretation	20 **YES page 8-10**	Give a cautious overall interpretation of results considering objectives, limitations, multiplicity of analyses, results from similar studies, and other relevant evidence
Generalisability	21 **YES Page8-10**	Discuss the generalisability (external validity) of the study results
**Other information**		
Funding	22 **YES Page 10**	Give the source of funding and the role of the funders for the present study and, if applicable, for the original study on which the present article is based

For peer review only - http://bmjopen.bmj.com/site/about/guidelines.xhtml

*Give information separately for cases and controls in case-control studies and, if applicable, for exposed and unexposed groups in cohort and cross-sectional studies.

Note: An Explanation and Elaboration article discusses each checklist item and gives methodological background and published examples of transparent reporting. The STROBE checklist is best used in conjunction with this article (freely available on the Web sites of PLoS Medicine at http://www.plosmedicine.org/, Annals of Internal Medicine at http://www.annals.org/, and Epidemiology at http://www.epidem.com/). Information on the STROBE Initiative is available at www.strobe-statement.org.

For peer review only

BMJ Open

Prevalence of high screen-time and associated factors among students: a cross-sectional study in Zhejiang, China

Journal:	*BMJ Open*
Manuscript ID	bmjopen-2018-021493.R1
Article Type:	Research
Date Submitted by the Author:	31-Mar-2018
Complete List of Authors:	Wang, Hao; Zhejiang Provincial Center for Diseases Control and Prevention, Department of NCDs Control and Prevention<br>Zhong, Jie-Ming; Zhejiang Provincial Center for Diseases Control and Prevention, Department of NCDs Control and Prevention<br>Hu, Ru-Ying; Zhejiang Provincial Center for Diseases Control and Prevention, Department of NCDs Control and Prevention<br>Bragg, Fiona; University of Oxford, Nuffield Department of Population Health<br>Yu, Min; Zhejiang Provincial Center for Diseases Control and Prevention, Department of NCDs Control and Prevention<br>Du, Huaidong; University of Oxford, Clinical Trial Service Unit & Epidemiological Studies Unit (CTSU), Nuffield Department of Population Health; University of Oxford, Medical Research Council Population Health Research Unit, Nuffield Department of Population Health
**Primary Subject Heading**:	Epidemiology
Secondary Subject Heading:	Epidemiology
Keywords:	Screen time, Behaviours, Lifestyle, Adolescents, Factors

SCHOLARONE™
Manuscripts

Prevalence of high screen-time and associated factors among students: a cross-sectional study in Zhejiang, China

Hao Wang[1], Jieming Zhong[1], Ruying Hu[1], Bragg Fiona[2], Min Yu[1], Huaidong Du[2, 3]

Author affiliations

1. Department of NCDs Control and Prevention, Zhejiang Provincial Center for Diseases Control and Prevention, Hangzhou, China

2. Clinical Trial Service Unit & Epidemiological Studies Unit (CTSU), Nuffield Department of Population Health, University of Oxford, Oxford, UK

3. Medical Research Council Population Health Research Unit, Nuffield Department of Population Health, University of Oxford, Oxford, UK

Correspondence to Min Yu; myu@cdc.zj.cn

For peer review only

For peer review only - http://bmjopen.bmj.com/site/about/guidelines.xhtml

Strengths and limitations of this study

- This is a school-based study with a representative sample from provincial China, a high response rate and a standardized procedure.
- The study questionnaire covers a range of socio-demographic and behavioral risk factors, and the findings provide evidence to support health, and other, professionals in formulating intervention strategies to control screen-time.
- The cross-sectional study design prevents establishment of causal relationships between socio-demographic and behavioural factors and high screen-time (ST).
- Detailed information about ST (such as duration of watching TV, computer use and playing electronic games) is not available in this study.

For peer review only - http://bmjopen.bmj.com/site/about/guidelines.xhtml

Abstract

Objective To investigate the prevalence and correlates of high screen-time (ST) among students in Zhejiang, China.

Design Cross-sectional study.

Setting School-based adolescent health survey in Zhejiang Province, China.

Participants 23 543 students in grades 7-12 from 442 different schools.

Outcome High screen-time (ST).

Results The mean age of the students was 15.6 years and 49.7% of them were girls. The prevalence of high ST (screen viewing ⩾2 hours per day) was 42.4% (95%CI: 40.2-44.5), higher in boys than in girls (45.4% [95%CI: 42.8-48.0] vs. 39.1% [95%CI: 36.6-41.7]). No statistically significant difference was found between urban and rural areas (43.0% [95%CI: 37.2-48.7] vs. 42.1% [95%CI: 39.6-44.6]). The prevalence of high ST among middle school, academic high school, and vocational high school students was 35.3%, 30.0% and 73.5%, respectively. Multivariable logistic analysis showed that older age, attendance at vocational high school, non-intact family, poor academic performance, bad self-reported health status, loneliness, and drinking carbonated beverages ⩾3 times every day were positively associated with high ST. Attendance at academic high school, higher parental education, and being physically active were negatively associated with high ST.

Conclusions High ST was prevalent among students and associated with a cluster of socio-demographic and behavioural risk factors in Zhejiang, China.

Summary Through a provincial representative survey, we found that excessive electronic screen product exposure appears to be an increasingly common behaviour among middle and high school students. The findings provide evidence to support the development and implementation of policies or interventions to control high ST among students in Zhejiang.

Keywords Screen-time; Behaviours; Lifestyle; Adolescents; Factors

For peer review only - http://bmjopen.bmj.com/site/about/guidelines.xhtml

For peer review only

For peer review only - http://bmjopen.bmj.com/site/about/guidelines.xhtml

Introduction

Rapid economic development over recent decades has been accompanied by dramatic transitions in lifestyles in China. Prolonged sedentary time and low physical activity are becoming more common in China,[1] and exposure to electronic screen products is known to be the most population sedentary leisure activity among adolescents. At the same time, prevalence of obesity and diabetes have increased dramatically in the Chinese population.[2-4]

According to a report released by China Internet Information Center,[5] the number of Internet users in China increased from 0.54 billion to 0.71 billion during 2012-2017, and the proportion of the population using mobile Internet devices increased from 72.2% to 92.5% over the same period. Compared with traditional desktop computers, smartphones provide an easier means for adolescents to access the Internet, resulting in more time spent on electronic screen products. In the United States, while the proportion of high school students exceeding the recommended 2 or fewer hours/day of television viewing time decreased significantly from 43% to 32% between 1999-2013, the proportion who spent more than 2 hours per day playing video or computer games nearly doubled from 22% to 41%.[6] This transition may also happen in China in the near future, where adolescents aged 10-19 years account for approximately 20% of the total population of China.[5]

In China, approximately 60% of inner-city adolescents are estimated to have one or more screen products in their bedrooms.[7] High screen-time (ST) among adolescents has been increasingly recognized as a serious public health concern and this continues into early adulthood.[8] Previous studies have indicated that high ST is not only associated with chronic diseases (e.g., obesity, metabolic syndrome),[9-12] but also with adolescents' psychological health.[13] One study found that watching television or using a computer for more than 3 hours a day was positively associated with health complaints (e.g., headache,

For peer review only

For peer review only - http://bmjopen.bmj.com/site/about/guidelines.xhtml

low mood, irritability and anxiety), and these associations were not mediated by low physical activity levels.[14] Another European study found a positive relationship between high ST and school problems (e.g., truancy and poor academic performance).[15] Evidence to-date on socio-demographic and lifestyle correlates of high ST comes mainly from western countries,[16-18] with little known about these associations in China. Although a previous Chinese study, including 5 003 adolescents found a high ST prevalence of 26%,[19] the study included only middle school, and not high school, students. The study was conducted in 2010, and, during the past seven years, technology has developed rapidly and screen products have become more widespread globally. It is important, therefore, to examine the prevalence of high ST and its correlating factors among adolescents in China.

Methods

Sample and procedure

A cross-sectional study was carried out between April and May 2017 in Zhejiang province, China, using a three-stage sampling design. In stage one, 30 counties, including 12 urban areas and 18 rural areas, were sampled randomly from all 90 counties in Zhejiang. In stage two, 10 classes of middle school, 5 classes of academic high school, and 5 classes of vocational high school were selected randomly within each chosen county. In stage three, all students attending the chosen classes were invited to participate in the survey. Written informed consent was obtained from all participants and their guardians before the survey. A total of 24 157 students from grades 7-12 in 442 different schools were invited to participate. A response rate of 97.5% was achieved, and after exclusion of individuals with missing or incomplete questionnaires, 23 543 participants were included in the final analyses. 12 068 (51.3%) were boys and the overall mean age was 15.6 years. 12 207 (51.9%) students were from middle schools, 6477 (27.5%) from academic high schools

For peer review only - http://bmjopen.bmj.com/site/about/guidelines.xhtml

and 4859 (20.6%) from vocational high schools. The survey questionnaire was based on the Youth Risk Behaviour Survey, developed by the Centers for Disease Control and Prevention (CDCs) [20] and the international Global School-based Student Health Survey supported by the World Health Organization.[21] The reliability of questionnaire has been reported in previous studies.[22-24] The questionnaire covered demographic characteristics, tobacco and alcohol use, physical activity, dietary habit, exposure to violence, injury, and sexual behaviours. The self-administrated questionnaire was filled anonymously by students and handed in directly to trained surveyors from local CDCs after completion. In order to improve response rate, every recruited student was given a pencil box as a gift. The study design and procedure was approved by the ethics committee of Zhejiang Provincial CDC.

Patient and public involvement

Study participants were generally healthy students and no patients were involved in the study. Students and their parents were not involved in the design and conduct of study. The findings will be disseminated to Department of Health and Department of Education in Zhejiang Province, but not directly to participating students.

Measures

Outcome variable: ST was assessed through the question: "On an average school day, how many hours do you watch television (TV), play pad or electronic games or use a smartphone or computer for something that is not school work? " (Answer options: "I do not watch TV, play pad or electronic games or use a smartphone or computer for something that is not school work", "<1h/d", "1h/d", "2h/d", "3h/d", "4h/d", and "≥5h/d"). Participants were considered as high screen-time users if they answered that they had watched a screen for more than 2 hours on an average school day.[25 26]

For peer review only - http://bmjopen.bmj.com/site/about/guidelines.xhtml

Main covariates: Information was collected on parental education level, parental marital status, academic performance, loneliness, physical activity, breakfast behaviour, and intake of fruit, vegetables and carbonated beverages (**Table 1**).

Statistical analysis

A weighting factor was applied to each student record to adjust for non-response and for the varying probabilities of selection. The weight used for estimation in this survey was given by: W= W1 $\times$W2 $\times$ f1 $\times$ f2, where W1= the inverse of the probability of selecting the county; W2= the inverse of the probability of selecting the classroom within the county; f1= a student-level nonresponse adjustment factor calculated by class; f2= a post-stratification adjustment factor calculated by grade.[27] Continuous variables were shown as mean ± standard deviation. Prevalence of high ST was estimated as percentage with its 95% confidence interval (CI). Between group comparisons of categorical variables were undertaken using the Chi-square test. Weighted prevalence between groups was calculated using the Rao-Scott chi-square test. Multivariable logistic regression was used to ascertain factors related with high ST. All analyses were performed with SAS software V.9.3. All statistical tests were two tailed, with *P*-values <0.05 considered statistically significant.

Results

Descriptive statistics

37.7% boys and 39.0% girls were from urban areas (**Table 2**). As compared to girls, boys were more likely to describe their personal health status as very good or good (56.0% vs.49.3%) and less likely to feel lonely (33.0% vs.38.7%). 16.7% of students reported being physically active every day. 70.6% of students reported consuming breakfast every day. 28.7% and 8.0% of students reported consuming fruit and vegetables, respectively,

For peer review only - http://bmjopen.bmj.com/site/about/guidelines.xhtml

less than once daily. There was no sex difference in the frequency of carbonated beverages consumption (*P*=0.19).

The prevalence of high ST

The overall prevalence of high ST was 42.4% (95%CI: 40.2-44.5), higher in boys than in girls (45.4% vs. 39.1%) (**Table 3**). There was no statistically significant difference in high ST prevalence between urban and rural areas (43.0% vs. 42.1%). Prevalence of high ST was positively associated with age (*P*<0.0001). The prevalence of high ST among students attending middle, academic high, and vocational high school was 35.3%, 30.0% and 73.5%, respectively.

Logistic regression analysis

After adjusting for all other socio-demographic and health-related behavior factors under investigation, parental education level was inversely associated with high ST (**Table 4**). Students whose fathers were educated to college level or above had 31% lower (OR=0.69, 95%CI: 0.58-0.82) risk of high ST compared with students whose fathers were educated to primary school level or below. Similar associations were seen for maternal education level; students whose mother were educated to college level or above had 34% lower risk of high ST (OR=0.66, 95%CI: 0.58-0.76) compared with students whose mothers were educated to primary school level or below. Students living in non-intact families had a 26% higher risk of high ST (OR=1.26, 95%CI: 1.13-1.41) than students living in intact families, and those with bad academic performance were 2.1 times more likely to report high ST than those with excellent academic performance (OR=2.07, 95%CI: 1.86-2.30). Compared with students with very good or good self-reported health, students with fair, bad or very bad self-reported health had 14% (OR=1.14, 95%CI: 1.07-1.22) and 31% (OR=1.31, 95%CI: 1.14-1.49) higher risk of high ST, respectively. Students who often or always felt lonely were 20% (OR=1.20, 95%CI: 1.08-1.34) more likely to report high ST than those

For peer review only - http://bmjopen.bmj.com/site/about/guidelines.xhtml

who never or occasionally felt lonely. Compared with students who were not physically active within the past 7 days, those who were physically active had 10% lower risk of high ST (OR=0.90, 95%CI: 0.81-0.99). Fruit consumption was not associated with ST in a linear association; compared with students who reported eating fruit 1-2 times per day, those who reported eating fruit less than one daily and ≥3 times per day had 44% (OR=1.44, 95%CI: 1.29-1.60) and 18% (OR=1.18, 95%CI: 1.01-1.38) higher risks of high ST, respectively. Compared with students who did not consume carbonated beverages, those who reported consuming carbonated beverages ⩾3 times per day had 29% higher odds of high ST (OR=1.29, 95%CI: 1.03-1.60).

Discussion

Through a provincial representative survey among students in Zhejiang, China, our study investigated the prevalence of high ST associated with several socio-demographic (e.g., parental education level and marital status) and behavioural factors (e.g., inadequate physical activity, skipping breakfast, insufficient intake of fruits, drinking carbonated beverages). The findings provide evidence to support the development and implementation of policies or interventions to control ST among middle and high school students in Zhejiang.

The prevalence of high ST

The use of different questionnaires to evaluate ST in this and previous studies makes direct comparison of the results difficult. In our study more than 40% students exceeded the recommended maximum ST of 2h/d, suggesting that excessive exposure to electronic screen products is becoming more common among adolescents in China. A study using a similar questionnaire as that used in the current study and conducted in Brazil in 2013-2014 showed that 59.5% of students aged 12-17 years were exposed to electronic screens for ≥ 2h/d,[28] higher than our study. Consistent with results from other studies, we

For peer review only

For peer review only - http://bmjopen.bmj.com/site/about/guidelines.xhtml

found that boys had higher prevalence of high ST than girls[7 19 29] and the prevalence of high ST increased with increasing age.[29 30] The gender difference might be explained by the fact that boys tend to be more attracted to computer games (such as sports, racing, fighting, shooting) than girls.[31] Another possible reason might be that girls usually spend more time on homework than boys in China. Notably, among students attending three different types of school, those attending vocational high schools had the highest prevalence of high ST, suggesting students of vocational high school may be an appropriate target population for interventions to reduce the prevalence of high ST. A possible explanation for differences between school types might be that vocational high school students do not face competitive high school or college entrance examinations, unlike students at middle and academic high schools. Hence, they might have more time available to spend on electronic screen products.

Relationship of high ST with its correlates

The inverse association between parental education level and high ST observed in our study may be due to the fact that highly-educated parents were more inclined to limit children's ST at home than less highly educated parents. In China, it is estimated that approximately 50% of families have no specific rules for ST,[7] but having ST rules at homes might have a protective effect on children's excessive ST.[7 32] In addition, students living in non-intact families and those often or always feeling lonely had much higher odds of high ST, which is consistent with previous studies.[33] These findings suggest parental care and company are important for this age group in terms of controlling high ST and improving academic performance, because academic performance was inversely associated with high ST, consistent with the findings of previous studies.[15 34] We found that poor self-reported health was positively associated with high ST. This might reflect bad health preventing students from engaging in physical activity, with an associated increased likelihood of excessive electronic screen product exposure. Another possible reason might

For peer review only - http://bmjopen.bmj.com/site/about/guidelines.xhtml

be that excessive electronic screen product exposure could have a negative impact on the health of adolescents.

As expected, being physically active was negatively associated with high ST in our study. A previous study found that junior school students in China lacked awareness of the importance of physical activity,[35] despite government guidelines suggesting adolescents should undertake at least 60 minutes of physical activity daily.[36] It is possible that reducing ST would eventually increase physical activity levels with associated psychological benefits and improved quality of life, academic performance and self-esteem.[37 38] Our study demonstrated that only about 16.7% students reported being physically active every day during the past 7 days, which was lower than the average level in China (22.7%),[39] suggesting action is needed to increase physical activity levels among adolescents in Zhejiang.

Dietary guidelines in China recommend consumption of 200-350 grams of fruit and 300-500 grams of vegetables daily. It is not possible to estimate exact quantities of fruit and vegetables consumed daily through a self-administrated survey among adolescents, but our study found over 70% of students consumed fruits once daily or more frequently, higher than the proportion (64%) among adolescents in the USA.[40] A previous study found television viewing was inversely related to intake of fruit (OR=0.92) and vegetables (OR=0.95) among the United States adolescents.[41] In our study, compared to students consuming fruits 1-2 times per day, those consuming fruits less than once a day had a higher likelihood of high ST. Interestingly, those consuming fruits ≥3 times per day also had a higher probability of high ST, in contrast with results from a previous study in the USA[41] in which fruit intake was divided into two groups ("≥1 time per day" and "<1 time per day"). One possible explanation for this U-shaped association might be that some adolescents with excessive exposure to electronic screen may consume excessive

For peer review only - http://bmjopen.bmj.com/site/about/guidelines.xhtml

quantities of fruit. In the present study, half of the students consumed vegetable ≥3 times every day, higher than the United States (18.5%).[42] Although vegetable intake was negatively associated with high ST in univariate logistic regression, there was no statistically significant association in multivariable analyses.

Many studies have demonstrated associations of sugar sweetened beverages (SSB) with chronic diseases (e.g., obesity, diabetes, hypertension, stroke).[43-46] Schulze et al. found that individuals consuming ≥1 SSB per day had an 83% higher risk of developing type 2 diabetes compared with those consuming < 1 SSB per month.[43] In our study, 6.9% of students reported consuming carbonated beverages ≥1 time per day. Although the percentage was far lower than the United States (20.4%),[47] this raises concerns about SSB consumption among adolescents in China. Gebremariam et al. found that an increase in TV viewing by an hour was associated with the consumption of 30 millilitre more soft drinks in Greece and 90 millilitre more soft drinks in Switzerland.[16] The present study showed that students consuming carbonated drinks ≥ 3 times per day have about 30% higher risk of high ST, which was consistent with a previous study.[48] It is possible that students with high ST might be more frequently exposed to food advertisements, which are often for unhealthy foods, including carbonated beverages.[49]

Implications

With the development of internet technology and the emergence of new screen products, it is inevitable that adolescents will have more opportunity to spend time using electronic screen products. Our study has several important implications. First, excessive electronic screen product exposure appears to be an increasingly common behaviour among students in Zhejiang, and without further intervention, it will become more common over coming decades. Second, comprehensive intervention measures, including strict rules on duration of using electronic products at home and increasing physical activity need to be

For peer review only - http://bmjopen.bmj.com/site/about/guidelines.xhtml

taken into account; these interventions might also benefit the development of healthy dietary habits, improving physiological and psychological health.

Strengths and limitations

The strengths of this study include the large provincially representative sample, high response rate, and use of standardized procedures. In addition, the study questionnaire included a large number of socio-demographic and behavioral risk factors. There are also, however, several limitations. First, the cross-sectional study design prevents establishment of the causal relationship of these factors with high ST. Second, the questionnaire focused on aggregated ST, and did not allow investigation of time spent on specific screen products (e.g., television, computer, video game, and mobile phone). Third, all data were self-reported by students and not objectively measured, which might increase the risk of information bias. Fourth, we only collected information on ST on school days, and did not include non-school days. Given that students usually spent more time on screen products on non-school days,[7] the prevalence of high ST observed in our study might be an underestimate.

Conclusions

In summary, our study extended existing literature by describing the patterns and associations of high ST among a provincial representative sample of adolescents in China, and found that high ST is prevalent among students and associated with a cluster of socio-demographic and unhealthy behavioural risk factors in Zhejiang China.

For peer review only - http://bmjopen.bmj.com/site/about/guidelines.xhtml

Acknowledgements

The authors would like to thank all the students, parents, teachers and local officials for their participation, assistance and co-operation.

Contributor HW and MY designed the study. HW collected and analyzed data and wrote manuscript. JZ and RH were involved in data interpretation. HD and BF provided critical comments on the manuscript. All the authors have read and approved the final submitted version.

Competing interests None declared.

Funding The work was supported by grant (2016YFC0900502) from National Key Research and Development Program of China.

Ethics approval Ethics Committee of Zhejiang Provincial Centre for Disease Control and Prevention.

Data sharing statement No additional data are available

For peer review only

For peer review only - http://bmjopen.bmj.com/site/about/guidelines.xhtml

References

1. Ng SW, Howard AG, Wang HJ, et al. The physical activity transition among adults in China: 1991-2011. *Obes Rev* 2014;15 Suppl 1:27-36. doi: 10.1111/obr.12127
2. Ji CY, Working Group on Obesity in C. Report on childhood obesity in China (4) prevalence and trends of overweight and obesity in Chinese urban school-age children and adolescents, 1985-2000. *Biomed Environ Sci* 2007;20(1):1-10.
3. Wang L, Gao P, Zhang M, et al. Prevalence and Ethnic Pattern of Diabetes and Prediabetes in China in 2013. *Jama* 2017;317(24):2515-23. doi: 10.1001/jama.2017.7596 [published Online First: 2017/06/28]
4. Trang NH, Hong TK, Dibley MJ. Cohort profile: Ho Chi Minh City Youth Cohort--changes in diet, physical activity, sedentary behaviour and relationship with overweight/obesity in adolescents. *BMJ Open* 2012;2(1):e000362. doi: 10.1136/bmjopen-2011-000362
5. Centre CINI. China Internet Development Statistics Report. Beijing, 2016.
6. Kann L, Kinchen S, Shanklin SL, et al. Youth risk behavior surveillance--United States, 2013. *MMWR Suppl* 2014;63(4):1-168.
7. Jiang XX, Hardy LL, Ding D, et al. Recreational screen-time among Chinese adolescents: a cross-sectional study. *J Epidemiol* 2014;24(5):397-403.
8. Busschaert C, Cardon G, Van Cauwenberg J, et al. Tracking and predictors of screen time from early adolescence to early adulthood: a 10-year follow-up study. *J Adolesc Health* 2015;56(4):440-8. doi: 10.1016/j.jadohealth.2014.11.016
9. Grontved A, Ried-Larsen M, Moller NC, et al. Youth screen-time behaviour is associated with cardiovascular risk in young adulthood: the European Youth Heart Study. *Eur J Prev Cardiol* 2014;21(1):49-56. doi: 10.1177/2047487312454760
10. Kang HT, Lee HR, Shim JY, et al. Association between screen time and metabolic syndrome in children and adolescents in Korea: the 2005 Korean National Health and Nutrition Examination Survey. *Diabetes Res Clin Pract* 2010;89(1):72-8. doi: 10.1016/j.diabres.2010.02.016
11. Robinson TN, Banda JA, Hale L, et al. Screen Media Exposure and Obesity in Children and Adolescents. *Pediatrics* 2017;140(Suppl 2):S97-S101. doi: 10.1542/peds.2016-1758K
12. Mark AE, Janssen I. Relationship between screen time and metabolic syndrome in adolescents. *J Public Health (Oxf)* 2008;30(2):153-60. doi: 10.1093/pubmed/fdn022
13. Page AS, Cooper AR, Griew P, et al. Children's screen viewing is related to psychological difficulties irrespective of physical activity. *Pediatrics* 2010;126(5):e1011-7. doi: 10.1542/peds.2010-1154
14. Brindova D, Veselska ZD, Klein D, et al. Is the association between screen-based behaviour and health complaints among adolescents moderated by physical activity? *Int J Public Health* 2015;60(2):139-45. doi: 10.1007/s00038-014-0627-x
15. Husarova D, Blinka L, Madarasova Geckova A, et al. Do sleeping habits mediate the association between time spent on digital devices and school problems in adolescence? *Eur J Public Health* 2017 doi: 10.1093/eurpub/ckx198
16. Gebremariam MK, Chinapaw MJ, Bringolf-Isler B, et al. Screen-based sedentary time: Association with soft drink consumption and the moderating effect of parental education in European children: The ENERGY study. *PLoS One* 2017;12(2):e0171537. doi: 10.1371/journal.pone.0171537
17. Lee EY, Hunter S, Leatherdale ST, et al. Sociodemographic correlates of physical activity and screen time among adolescents in Canada and Guatemala: Results from the COMPASS system. *Glob Health Promot* 2017:1757975917710804. doi: 10.1177/1757975917710804
18. Brunetti VC, O'Loughlin EK, O'Loughlin J, et al. Screen and nonscreen sedentary behavior and sleep in adolescents. *Sleep Health* 2016;2(4):335-40. doi: 10.1016/j.sleh.2016.09.004
19. Cao H, Qian Q, Weng T, et al. Screen time, physical activity and mental health among urban adolescents in China. *Prev Med* 2011;53(4-5):316-20. doi: 10.1016/j.ypmed.2011.09.002
20. Centers for Disease Control and Prevention . 2017 State and Local Youth Risk Behavior Survey; 2017 [Available from: https://www.cdc.gov/healthyyouth/data/yrbs/questionnaires.htm accessed 21/11 2017.

For peer review only

For peer review only - http://bmjopen.bmj.com/site/about/guidelines.xhtml

21. World Health Organization.Global school-based students health survey(GSHS) 2015 [updated 7/6. Available from: http://www.who.int/ncds/surveillance/gshs/nepal/en/ accessed 21/11 2017.
22. Brener ND, Kann L, McManus T, et al. Reliability of the 1999 youth risk behavior survey questionnaire. *J Adolesc Health* 2002;31(4):336-42.
23. Zullig KJ, Pun S, Patton JM, et al. Reliability of the 2005 middle school Youth Risk Behavior Survey. *J Adolesc Health* 2006;39(6):856-60. doi: 10.1016/j.jadohealth.2006.07.008
24. Centers for Disease C, Prevention, Brener ND, et al. Methodology of the Youth Risk Behavior Surveillance System--2013. *MMWR Recomm Rep* 2013;62(RR-1):1-20.
25. American Academy of Pediatrics. Committee on Public E. American Academy of Pediatrics: Children, adolescents, and television. *Pediatrics* 2001;107(2):423-6.
26. Barlow SE, Expert C. Expert committee recommendations regarding the prevention, assessment, and treatment of child and adolescent overweight and obesity: summary report. *Pediatrics* 2007;120 Suppl 4:S164-92. doi: 10.1542/peds.2007-2329C
27. Pengpid S, Peltzer K. Alcohol use and associated factors among adolescent students in Thailand. *West Indian Med J* 2012;61(9):890-6.
28. Cureau FV, Ekelund U, Bloch KV, et al. Does body mass index modify the association between physical activity and screen time with cardiometabolic risk factors in adolescents? Findings from a country-wide survey. *Int J Obes (Lond)* 2017;41(4):551-59. doi: 10.1038/ijo.2016.210
29. Nguyen PV, Hong TK, Nguyen DT, et al. Excessive screen viewing time by adolescents and body fatness in a developing country: Vietnam. *Asia Pac J Clin Nutr* 2016;25(1):174-83. doi: 10.6133/apjcn.2016.25.1.21
30. Hoyos Cillero I, Jago R, Sebire S. Individual and social predictors of screen-viewing among Spanish school children. *Eur J Pediatr* 2011;170(1):93-102. doi: 10.1007/s00431-010-1276-6
31. Hartmann T, Klimmt C. Gender and Computer Games: Exploring Females' Dislikes. *Journal of Computer-Mediated Communication* 2006;11(4): 910–31 doi: 10.1111/j.1083-6101.2006.00301.x
32. Brindova D, Pavelka J, Sevcikova A, et al. How parents can affect excessive spending of time on screen-based activities. *BMC Public Health* 2014;14:1261. doi: 10.1186/1471-2458-14-1261
33. Wu X, Tao S, Zhang Y, et al. Low physical activity and high screen time can increase the risks of mental health problems and poor sleep quality among Chinese college students. *PLoS One* 2015;10(3):e0119607. doi: 10.1371/journal.pone.0119607
34. Yan H, Zhang R, Oniffrey TM, et al. Associations among Screen Time and Unhealthy Behaviors, Academic Performance, and Well-Being in Chinese Adolescents. *Int J Environ Res Public Health* 2017;14(6) doi: 10.3390/ijerph14060596
35. Ren YJ, Liu QM, Lu J, et al. [Behavior and knowledge on physical activity among urban junior students in Hangzhou]. *Zhonghua Liu Xing Bing Xue Za Zhi* 2012;33(7):672-5.
36. China National Education. Suggestion of CPC Central Committee and State Council on strengthening the youth sports to enhance youth physical. *Chin J Sch Health* 2007;28(6):481-83.
37. Suchert V, Hanewinkel R, Isensee B. Sedentary behavior and indicators of mental health in school-aged children and adolescents: A systematic review. *Prev Med* 2015;76:48-57. doi: 10.1016/j.ypmed.2015.03.026
38. Tremblay MS, Leblanc AG, Janssen I, et al. Canadian sedentary behaviour guidelines for children and youth. *Appl Physiol Nutr Metab* 2011;36(1):59-64; 65-71. doi: 10.1139/H11-012
39. Zhang X, Song Y, Yang TB, et al. [Analysis of current situation of physical activity and influencing factors in Chinese primary and middle school students in 2010]. *Zhonghua Yu Fang Yi Xue Za Zhi* 2012;46(9):781-8.
40. Eaton DK, Kann L, Kinchen S, et al. Youth risk behavior surveillance - United States, 2011. *MMWR Surveill Summ* 2012;61(4):1-162.
41. Lipsky LM, Iannotti RJ. Associations of television viewing with eating behaviors in the 2009 Health Behaviour in School-aged Children Study. *Arch Pediatr Adolesc Med* 2012;166(5):465-72. doi: 10.1001/archpediatrics.2011.1407
42. Lowry R, Michael S, Demissie Z, et al. Associations of Physical Activity and Sedentary Behaviors with Dietary Behaviors among US High School Students. *J Obes* 2015;2015:876524. doi: 10.1155/2015/876524

For peer review only - http://bmjopen.bmj.com/site/about/guidelines.xhtml

43. Schulze MB, Manson JE, Ludwig DS, et al. Sugar-sweetened beverages, weight gain, and incidence of type 2 diabetes in young and middle-aged women. *JAMA* 2004;292(8):927-34. doi: 10.1001/jama.292.8.927
44. Palmer JR, Boggs DA, Krishnan S, et al. Sugar-sweetened beverages and incidence of type 2 diabetes mellitus in African American women. *Arch Intern Med* 2008;168(14):1487-92. doi: 10.1001/archinte.168.14.1487
45. Dhingra R, Sullivan L, Jacques PF, et al. Soft drink consumption and risk of developing cardiometabolic risk factors and the metabolic syndrome in middle-aged adults in the community. *Circulation* 2007;116(5):480-8. doi: 10.1161/CIRCULATIONAHA.107.689935
46. Fung TT, Malik V, Rexrode KM, et al. Sweetened beverage consumption and risk of coronary heart disease in women. *Am J Clin Nutr* 2009;89(4):1037-42. doi: 10.3945/ajcn.2008.27140
47. Kann L, McManus T, Harris WA, et al. Youth Risk Behavior Surveillance - United States, 2015. *MMWR Surveill Summ* 2016;65(6):1-174. doi: 10.15585/mmwr.ss6506a1
48. Kelishadi R, Mozafarian N, Qorbani M, et al. Association between screen time and snack consumption in children and adolescents: The CASPIAN-IV study. *J Pediatr Endocrinol Metab* 2017;30(2):211-19. doi: 10.1515/jpem-2016-0312
49. Li D, Wang T, Cheng Y, et al. The extent and nature of television food advertising to children in Xi'an, China. *BMC Public Health* 2016;16(1):770. doi: 10.1186/s12889-016-3468-0

For peer review only

For peer review only - http://bmjopen.bmj.com/site/about/guidelines.xhtml

Table 1. Questions and answer options included in the survey

Variables	Questions	Answer options
Parental education level	What is the highest level of education your father/mother has obtained? (separately for father and mother）	Primary school or below, Middle school, High school, College or university, Master graduates or above, Unknown.
Parental marital status	What is your parents current marital status?	Married, Divorced, Widowed, Separated.
Academic performance	How would you describe your grades in your class?	Excellent, Middle, Poor.
Self-reported health	In general, how would you describe your health status?	Very good, Good, Fair, Bad, Very bad, and Unknown
Loneliness	During the past 12 months, did you ever feel lonely?	Never, Occasionally, Sometimes, Often, Always.
Physical activity	During the past 7 days, on how many days were you physically active for a total of at least 60 minutes per day?	None, 1 day, 2 days, 3 days, 4 days, 5 days, 6 days, 7 days.
Breakfast	During the past 7 days, on how many days did you eat breakfast?	0 days, 1 day, 2 days, 3 days, 4 days, 5 days, 6 days, 7 days.
Fruit	During the past 30 days, how many times per day did you usually eat fruit, such as apples, oranges, mangoes, or papayas?	None, < 1 time/d, 1 time/d, 2 times/d, 3 times/d, 4 times /d, ≥ 5 times/d.
Vegetable	During the past 30 days, how many times per day did you usually eat vegetables, such as cauliflower, cabbage?	None, < 1 time/d, 1 time/d, 2 times/d, 3 times/d, 4 times/d, ≥ 5 times/d.
Carbonated beverages	During the past 30 days, how many times per day did you usually drink carbonated soft drinks, such as Coca-Cola, Pepsi, or Sprite? (Do not include diet soft drinks.)	None, < 1 times/d, 1 time/d, 2 times/d, 3 times/d, 4 times/d, ≥ 5 times/d.

For peer review only - http://bmjopen.bmj.com/site/about/guidelines.xhtml

Table 2. Characteristics of students from Zhejiang

Characteristics	Total (N=23 543)	Boys (N=12 068, 51.3%)	Girls (N=11 475, 49.7%)	*P*
Age (years)				0.577
≤13	5159 (21.9)	2689 (22.3)	2470 (21.5)	
14	4300 (18.3)	2192 (18.1)	2108 (18.4)	
15	3730 (15.8)	1905 (15.8)	1825 (15.9)	
≥16	10 354 (44.0)	5282 (43.8)	5072 (44.2)	
Area				0.031
Urban	9022 (38.3)	4544 (37.7)	4478 (39.0)	
Rural	14 521 (61.7)	7524 (62.3)	6997 (61.0)	
Types of school				0.008
Middle school	12 207 (51.8)	6364 (52.7)	5843 (50.9)	
Academic high school	6477 (27.5)	3223 (26.7)	3254 (28.4)	
Vocational high school	4859 (20.6)	2481 (20.6)	2378 (20.7)	
Paternal education				0.0136
Primary or below	13 568 (57.6)	6908 (57.2)	6660 (58.0)	
Middle or high school	5100 (21.7)	2628 (21.8)	2472 (21.5)	
College or above	3129 (13.3)	1575 (13.1)	1554 (13.5)	
Unknown	1746 (7.4)	957 (7.9)	789 (7.0)	
Maternal education				<.0001
Primary or below	14 530 (61.7)	7292 (60.4)	7238 (63.1)	
Middle or high school	4363 (18.5)	2271 (18.8)	2092 (18.2)	
College or above	2736 (11.6)	1392 (11.5)	1344 (11.7)	
Unknown	1914 (8.1)	1113 (9.2)	801 (7.0)	
Parental marital status				0.0004
Married	21 151 (89.8)	10 924 (90.5)	10 227 (89.1)	
Other	2392 (10.2)	1144 (9.5)	1 248 (10.9)	
Academic performance				<.0001
Excellent	5448 (23.1)	2731 (22.6)	2717 (23.7)	
Middle	11 765 (50.0)	5727 (47.5)	6038 (52.6)	
Poor	6330 (26.9)	3610 (29.9)	2720 (23.7)	
Self-reported health				<.0001
Very good/good	12 415 (52.7)	6758 (56.0)	5657 (49.3)	
Fair	9563 (40.6)	4495 (37.2)	5068 (44.2)	
Very bad/bad	1293 (5.5)	650 (5.4)	643 (5.6)	
Unknown	272 (1.2)	165 (1.4)	107 (0.9)	
Loneliness				<.0001
Never/Occasionally	15 122 (64.2)	8082 (67.0)	7040 (61.3)	
Sometimes	5783 (24.6)	2698 (22.4)	3085 (26.9)	
Often/Always	2638 (11.2)	1288 (10.6)	1350 (11.8)	
Physical activity (d/wk)				<.0001
0	4883 (20.7)	2079 (17.2)	2804 (24.5)	
1-2	5690 (24.2)	2703 (22.4)	2987 (26.0)	
3-5	8050 (34.2)	4237 (35.1)	3813 (33.2)	
6-7	4920 (20.9)	3049 (25.3)	1871 (16.3)	
Breakfast (d/wk)				<.0001
0	473 (2.0)	289 (2.39)	184 (1.60)	
1-2	599 (2.5)	298 (2.47)	301 (2.62)	
3-4	1249 (5.3)	667 (5.53)	582 (5.07)	

For peer review only

For peer review only - http://bmjopen.bmj.com/site/about/guidelines.xhtml

≥5	21 222 (90.2)	10 814 (89.6)	10 408 (90.7)	
Fruit (times/d)				0.01
≥3	6847 (29.1)	3453 (28.6)	3394 (29.6)	
1-2	9945 (42.2)	5213 (43.2)	4732 (41.2)	
<1	6751 (28.7)	3402 (28.2)	3349 (29.2)	
Vegetable (times/d)				0.40
≥3	11 775 (50.0)	5984 (49.6)	5791 (50.5)	
1-2	9884 (42.0)	5108 (42.3)	4776 (41.6)	
<1	1884 (8.0)	976 (8.1)	908 (7.9)	
Carbonated beverages				0.19
None	9133 (38.8)	4717 (39.1)	4416 (38.5)	
1-6 times/wk	12 792 (54.3)	6529 (54.1)	6263 (54.6)	
1-2 times/d	1146 (4.9)	600 (5.0)	546 (4.7)	
≥3 times/d	472 (2.0)	222 (1.8)	250 (2.2)	

For peer review only

For peer review only - http://bmjopen.bmj.com/site/about/guidelines.xhtml

Table 3 Weighted prevalence of high screen-time among students from Zhejiang by different characteristics

Characteristics	Prevalence (%)*	Rao-Scott chi-square	*P*
Age (y)		89.05	<.0001
≤13	31.0 (27.4-34.7)		
14	36.4 (33.6-39.3)		
15	43.5 (40.2-46.9)		
≥16	49.6 (46.0-53.1)		
Sex		18.03	<.0001
Boys	45.4 (42.8-48.0)		
Girls	39.1 (36.6-41.7)		
Areas		0.06	0.81
Urban	43.0 (37.2-48.7)		
Rural	42.1 (39.6-44.6)		
Types of school		404.57	<.0001
Middle school	35.3 (33.0-37.6)		
Academic high school	30.0 (27.2-32.9)		
Vocational high school	73.5 (69.5-77.5)		

*Based on the weighted data.

For peer review only

Table 4. Crude and adjusted odd ratios for high screen-time in relation to socio-demographic and behavioural factors among students from Zhejiang

Characteristics	COR(95%CI)	AOR(95%CI)[a]
Age (ref: ≤13 y)		
14 y	1.27 (1.06-1.54)*	1.24 (1.05-1.46)*
15 y	1.71 (1.38-2.13)***	1.41 (1.16-1.71)**
≥16 y	2.18 (1.76-2.72)***	1.34 (1.07-1.69) *
Rural (ref: urban)	0.97 (0.73-1.27)	0.99 (0.85-1.15)
Types of school (ref: middle school)		
Academic high school	0.79 (0.67-0.93)*	0.61 (0.49-0.77)***
Vocational high school	5.09 (4.05-6.40)***	3.71 (2.83-4.85)***
Parental marital status (ref: married)		
Others	1.52 (1.37-1.69)***	1.26 (1.13-1.41) ***
Paternal education (ref: primary or below)		
Middle or high school	0.75 (0.68-0.82)***	0.88 (0.81-0.98)*
College or above	0.40 (0.33-0.48)***	0.69 (0.58-0.82)***
Unknown	0.98 (0.85-1.13)	0.83 (0.68-1.01)
Maternal education (ref: primary or below)		
Middle or high school	0.75 (0.67-0.83)***	0.90 (0.82-1.00)
College or above	0.38 (0.33-0.45)***	0.66 (0.58-0.76)***
Unknown	1.14 (1.00-1.30)*	1.18 (0.99-1.41)
Academic performance (ref: excellent)		
Middle	1.55 (1.42-1.69)***	1.36 (1.25-1.48)***
Poor	2.35 (2.12-2.60)***	2.07 (1.86-2.30)***
Self-reported health (ref: very good/good)		
Fair	1.39 (1.30-1.49)***	1.14 (1.07-1.22)***
Bad/very bad	1.59 (1.38-1.84)***	1.31 (1.14-1.49)***
Unknown	1.46 (1.07-2.00)*	1.11 (0.78-1.57)
Loneliness (ref: never/occasionally)		
Sometimes	1.30 (1.20-1.40)***	1.20 (1.09-1.32)***
Often/Always	1.38 (1.24-1.54)***	1.20 (1.08-1.34)**
Physical activity (ref: 0 d/wk)		
1-2 d/wk	0.81 (0.74-0.89)***	0.90 (0.81-0.99)*
3-5 d/wk	0.74 (0.68-0.82)***	0.91 (0.83-0.99)*
6-7 d/wk	0.69 (0.61-0.79)***	0.93 (0.82-1.05)
Breakfast (ref: 0 d/wk)		
1-2 d/wk	1.38 (1.03-1.85)*	1.25 (0.92-1.70)
3-4 d/wk	1.21 (0.97-1.51)	1.04 (0.81-1.33)
≥5 d/wk	0.70 (0.57-0.86)**	0.95 (0.74-1.22)
Fruit (ref: 1-2 times/d)		
<1 times/d	2.36 (2.09-2.66)***	1.44 (1.29-1.60)***
≥3 times/d	2.01 (1.65-2.44)***	1.18 (1.01-1.38)*
Vegetable (ref: 1-2 times/d)		
<1 times/d	1.28 (1.13-1.45)***	0.99 (0.86-1.14)
≥3 times/d	1.27 (1.16-1.39)***	0.98 (0.90-1.07)
Carbonated beverages (ref: none)		
1-6 times/wk	1.02 (0.95-1.09)	1.02 (0.96-1.09)
1-2 times/d	0.98 (0.83-1.15)	0.99 (0.84-1.18)
≥3 times/d	1.50 (1.25-1.80)***	1.29 (1.03-1.60)*

COR: Crude odds ratio. AOR: Adjusted odds ratios. CI: confidence intervals. *: *P*<0.05. **: *P*<0.001. *** *P*<0.0001
a. Adjusted for all other covariates listed in the table.

For peer review only - http://bmjopen.bmj.com/site/about/guidelines.xhtml

STROBE Statement—checklist of items that should be included in reports of observational studies

	Item No	Recommendation
**Title and abstract**	1 **YES Title:page1 Abstract: page3**	(*a*) Indicate the study's design with a commonly used term in the title or the abstract
		(*b*) Provide in the abstract an informative and balanced summary of what was done and what was found
**Introduction**		
Background/rationale	2 **YES Page4**	Explain the scientific background and rationale for the investigation being reported
Objectives	3 **YES Page 3**	State specific objectives, including any prespecified hypotheses
**Methods**		
Study design	4 **YES Page5-6**	Present key elements of study design early in the paper
Setting	5 **YES Page4-6**	Describe the setting, locations, and relevant dates, including periods of recruitment, exposure, follow-up, and data collection
Participants	6 **YES Page4-5**	(*a*) *Cohort study*—Give the eligibility criteria, and the sources and methods of selection of participants. Describe methods of follow-up<br>*Case-control study*—Give the eligibility criteria, and the sources and methods of case ascertainment and control selection. Give the rationale for the choice of cases and controls<br>*Cross-sectional study*—Give the eligibility criteria, and the sources and methods of selection of participants
		(*b*) *Cohort study*—For matched studies, give matching criteria and number of exposed and unexposed<br>*Case-control study*—For matched studies, give matching criteria and the number of controls per case
Variables	7 **YES Page6, 15**	Clearly define all outcomes, exposures, predictors, potential confounders, and effect modifiers. Give diagnostic criteria, if applicable
Data sources/ measurement	8* **YES Page 5, 15**	For each variable of interest, give sources of data and details of methods of assessment (measurement). Describe comparability of assessment methods if there is more than one group
Bias	9 **YES Page 5**	Describe any efforts to address potential sources of bias
Study size	10 **YES Page4-5**	Explain how the study size was arrived at
Quantitative variables	11 **YES Page5-6**	Explain how quantitative variables were handled in the analyses. If applicable, describe which groupings were chosen and why
Statistical methods	12 **YES Page 7**	(*a*) Describe all statistical methods, including those used to control for confounding
		(*b*) Describe any methods used to examine subgroups and interactions
		(*c*) Explain how missing data were addressed
		(*d*) *Cohort study*—If applicable, explain how loss to follow-up was addressed<br>*Case-control study*—If applicable, explain how matching of cases and

For peer review only

		controls was addressed<br>*Cross-sectional study*—If applicable, describe analytical methods taking account of sampling strategy
		(*e*) Describe any sensitivity analyses
**Results**		
Participants	13*<br>**YES**<br>**Page 5**	(a) Report numbers of individuals at each stage of study—eg numbers potentially eligible, examined for eligibility, confirmed eligible, included in the study, completing follow-up, and analysed
		(b) Give reasons for non-participation at each stage
		(c) Consider use of a flow diagram
Descriptive data	14*<br>**YES**<br>**Page 19-20**	(a) Give characteristics of study participants (eg demographic, clinical, social) and information on exposures and potential confounders
		(b) Indicate number of participants with missing data for each variable of interest
		(c) *Cohort study*—Summarise follow-up time (eg, average and total amount)
Outcome data	15*<br>**YES**<br>**Page 6, 21**	*Cohort study*—Report numbers of outcome events or summary measures over time
		Case-control study—Report numbers in each exposure category, or summary measures of exposure
		Cross-sectional study—Report numbers of outcome events or summary measures
Main results	16<br>**YES**<br>**Page22-23**	(*a*) Give unadjusted estimates and, if applicable, confounder-adjusted estimates and their precision (eg, 95% confidence interval). Make clear which confounders were adjusted for and why they were included
		(*b*) Report category boundaries when continuous variables were categorized
		(*c*) If relevant, consider translating estimates of relative risk into absolute risk for a meaningful time period
Other analyses	17<br>**No**	Report other analyses done—eg analyses of subgroups and interactions, and sensitivity analyses
**Discussion**		
Key results	18 **YES**<br>**Page 7-9**	Summarise key results with reference to study objectives
Limitations	19<br>**YES**<br>**Page 13**	Discuss limitations of the study, taking into account sources of potential bias or imprecision. Discuss both direction and magnitude of any potential bias
Interpretation	20 **YES page 9-12**	Give a cautious overall interpretation of results considering objectives, limitations, multiplicity of analyses, results from similar studies, and other relevant evidence
Generalisability	21 **YES**<br>**Page12**	Discuss the generalisability (external validity) of the study results
**Other information**		
Funding	22 **YES**<br>**Page 14**	Give the source of funding and the role of the funders for the present study and, if applicable, for the original study on which the present article is based

For peer review only

For peer review only - http://bmjopen.bmj.com/site/about/guidelines.xhtml

*Give information separately for cases and controls in case-control studies and, if applicable, for exposed and unexposed groups in cohort and cross-sectional studies.

Note: An Explanation and Elaboration article discusses each checklist item and gives methodological background and published examples of transparent reporting. The STROBE checklist is best used in conjunction with this article (freely available on the Web sites of PLoS Medicine at http://www.plosmedicine.org/, Annals of Internal Medicine at http://www.annals.org/, and Epidemiology at http://www.epidem.com/). Information on the STROBE Initiative is available at www.strobe-statement.org.

For peer review only
